# Letter to the editor “Middle trapezius transfer for treatment of irreparable supraspinatus tendon tears-anatomical feasibility study”

**DOI:** 10.1186/s40634-021-00355-w

**Published:** 2021-04-29

**Authors:** Mehmet Kapicioglu, Orkhan Aliyev, Kerem Bilsel

**Affiliations:** Department of Orthopaedics and Traumatology, Bezmialem Vakıf University, Istanbul, Turkey

We read with great interest the recent original article entitled ‘Middle trapezius transfer for treatment of irreparable supraspinatus tendon tears-anatomical feasibility study’ by Moroder et al. [2]. We congratulate the authors for their interesting study that investigated the anatomical feasibility of a middle trapezius transfer below the acromion to replace the former supraspinatus unit for treating irreparable supraspinatus tendon tears. They determined that transferring the acromial portion of the middle trapezius to replace an irreparable supraspinatus was feasible in terms of size, vector, excursion, mobility, and safety. Although they approached a very pertinent topic in a scientific manner, we would like to ask the authors to clarify two concerns.

First, the authors mentioned that transfer of the acromial portion of the middle trapezius is a feasible replacement for the superior aspect of the rotator cuff, which mimics its features in terms of tendon dimensions and force vector. However, it may not support the depressing power of the supraspinatus and infraspinatus muscles in the inferior direction of the humeral head. It might even weaken the deltoid power by pulling the humeral head towards the acromion due to the superior direction of the force vector. Could this cause future pseudoparalysis and rupture and deltoid weakening with subacromial impingement with superior traction?

Second, as mentioned in the article, there is concern regarding the sufficiency of transfer excursion as scapula protraction can increase the pathway length of the transfer, and the tendon length seems to be one of the main factors affecting the tendon transfer negatively. Bassem et al. used an Achilles allograft to ensure the tendon length in lower trapezius transfer, [1] while you suggest hamstring grafts as another option [3]. What do you prefer in these situations?

We think that these two topics need to be investigated. Therefore, we would like to ask to the authors for their thoughts and opinions. Please let us know if the authors have any ideas regarding our questions.
